# Diseases of the Ear

**Published:** 1905-09

**Authors:** 


**Diseases of the Ear.** By James Kerr Love, M.D. Pp. xvi., 339. Bristol: John Wright & Co. 1904.

The *raison d'être* of this book would appear to be the series of stereograms illustrative of the anatomy of the temporal bone exhibited at the end, for although a large, expensive, and attractive volume has been produced, apart from these there is nothing new, and the letterpress does not contain more reading than some of the quite small manuals. We are inclined to think that it would have been better had the author brought out these in the form of a small atlas instead of adding another expensive text-book to those already in the field. Useful as these stereograms are, the only way for him who would operate on the temporal bone with satisfaction to himself and safety to the patient is to chip away at the bone itself. We must say that the book is most lucidly and delightfully written, the general get-up excellent, and many of the stereograms very beautiful. The first chapter deals with anatomy and physiology, the description of the internal ear being more easily explained than in any anatomical text-book. Under the physiology is a dissertation on the theory of harmony, which appears to us quite out of place in a work of this kind. Under diagrams and methods of examination all the regular means at our disposal are clearly described. Prognosis is considered under two headings, (1) Hearing and (2) Danger to Life. Treatment under three headings, viz. (1) The Prevention of Disease, (2) The Management of Deafness not Associated with Ear Discharge, (3) The Management of Suppurative Disease within the Temporal Bone. The description of the diseases of the ear are taken in the usual order, those of the middle ear, non-suppurative and suppurative alike, being dealt with in one chapter—the former, we think, too briefly, the latter, with its complications, at much greater length, forming the best and indeed the greater part of the book. The author endeavours to impress upon the reader the importance of following cases of suppuration to a satisfactory termination, viz. cure. We cannot congratulate the author on the coloured plates of the drum—those representing the normal condition are distinctly poor. The book ends with a chapter on deaf mutes and their education, a subject in which the author has had a large amount of experience, and which he deals with freely and well with a number of illustrative cases.

